# Photodynamic Antimicrobial Polymers for Infection Control

**DOI:** 10.1371/journal.pone.0108500

**Published:** 2014-09-24

**Authors:** Colin P. McCoy, Edward J. O’Neil, John F. Cowley, Louise Carson, Áine T. De Baróid, Greg T. Gdowski, Sean P. Gorman, David S. Jones

**Affiliations:** 1 Queen’s University Belfast, School of Pharmacy, Belfast, United Kingdom; 2 Blue Highway, Inc., Center for Science & Technology, Syracuse University, Syracuse, New York, United States of America; MGH, MMS, United States of America

## Abstract

Hospital-acquired infections pose both a major risk to patient wellbeing and an economic burden on global healthcare systems, with the problem compounded by the emergence of multidrug resistant and biocide tolerant bacterial pathogens. Many inanimate surfaces can act as a reservoir for infection, and adequate disinfection is difficult to achieve and requires direct intervention. In this study we demonstrate the preparation and performance of materials with inherent photodynamic, surface-active, persistent antimicrobial properties through the incorporation of photosensitizers into high density poly(ethylene) (HDPE) using hot-melt extrusion, which require no external intervention except a source of visible light. Our aim is to prevent bacterial adherence to these surfaces and eliminate them as reservoirs of nosocomial pathogens, thus presenting a valuable advance in infection control. A two-layer system with one layer comprising photosensitizer-incorporated HDPE, and one layer comprising HDPE alone is also described to demonstrate the versatility of our approach. The photosensitizer-incorporated materials are capable of reducing the adherence of viable bacteria by up to 3.62 Log colony forming units (CFU) per square centimeter of material surface for methicillin resistant *Staphylococcus aureus* (MRSA), and by up to 1.51 Log CFU/cm^2^ for *Escherichia coli*. Potential applications for the technology are in antimicrobial coatings for, or materials comprising objects, such as tubing, collection bags, handrails, finger-plates on hospital doors, or medical equipment found in the healthcare setting.

## Introduction

Hospital-acquired (nosocomial) infections pose a global healthcare concern. It has been estimated that 1 in 10 patients will acquire an infection after admission to a healthcare institution [1]. Such infection presents a serious risk to the morbidity and mortality of the most vulnerable individuals, who are being cared for in the very environment where recuperation and improvement in health and wellbeing is intended. The financial burden to healthcare systems is also alarming; a decade ago the annual economic cost of nosocomial infection in the US was determined to be in the region of $6.7 billion [1]. The costs of implementing strategies to prevent nosocomial infection is likely much less than the value of resources consumed in treatment of these infections once they occur [2]. A major concern in the treatment of nosocomial infection is the emergence of bacterial pathogens displaying resistance to a broad range of antibacterial chemotherapeutic drugs [3]. While the discovery of antibiotics has proved one of the most important advances in healthcare in the 20^th^ century, their widespread use is a double-edged sword. There has been a profound effect on the selective adaptation of bacteria, with multi-drug resistant strains (MDR) emerging at an alarming rate, and threatening the end of the “antibiotic era” [4], [5]. Antibiotic resistant bacteria pose a serious problem in hospitals; strains of methicillin resistant *Staphylococcus aureus* (MRSA) appear well adapted to the healthcare environment and have spread internationally (epidemic MRSA, EMRSA) [6]. With the discovery of the next generation of new and efficacious antibiotics lagging behind the emergence of MDR bacteria, the importance of hygiene and disinfection practices in healthcare institutions requires particular emphasis; such interventions may prevent cross-colonization of patients due to contamination of inanimate objects, such as handrails, bedding and medical equipment, or even the skin of healthcare workers and patients, acting as a reservoir for nosocomial infection [7]. In addition to MRSA, there is evidence to suggest that other pathogens may be transmitted by means of environmental reservoirs, including viral pathogens (influenza virus, norovirus, hepatitis, coronavirus), and problematic Gram-negative pathogens (*Escherichia coli*, *Clostridium difficile*, *Pseudomonas aeruginosa*, vancomycin-resistant enterococci) [7]. The present mainstay employed in controlling hospital infection is cleansing using biocides (antiseptics and disinfectants), such as quaternary ammonium compounds (QACs), halogen-releasing agents, and phenolics. The activity of such biocide agents depends on several factors, most notably concentration, period of contact, pH, temperature, numbers and nature of microorganisms to be inactivated [8]. There is also concern that intensive exposure of nosocomial pathogens to biocides may allow for the selection of biocide resistant/tolerant bacterial strains [8]–[12]. Unlike antibiotics that act at a specific cellular target or interfere with a defined metabolic process, biocides act in a non-specific manner at a variety of cellular targets, such as the bacterial outer membrane or cell wall, the cytoplasmic membrane, proteins, genetic material, and other cytosolic components [13]. Despite this, MRSA strains that show resistance to antiseptics and disinfectants have been isolated from clinical samples, with resistance due to the presence of genes encoding for energy-dependent drug efflux mechanisms, and these genes also confer cross-resistance to a diverse range of antimicrobial drugs [10]. In addition to acquired genetic resistance, bacteria in the biofilm mode of growth are inherently tolerant to inactivation using biocides [14]. Bacterial biofilms are defined as a sessile community of bacteria, characterized by cells that are irreversibly attached to a substratum or interface, or to each other, and are imbedded in a matrix of extracellular polymeric substances (EPS) that they have produced, and exhibit an altered phenotype with respect to growth rate and gene transcription [15]. The biofilm provides an environment where antimicrobial penetration is hindered, genetic exchange and resistance transfer are facilitated, and a change in physiological state, such as stationary phase dormant zones, are a significant factor in the resistance to antibacterial challenge [16], [17]. Numerous studies have demonstrated the difficulties in biofilm eradication using biocides commonly employed for cleansing purposes in the hospital setting [18]–[21]. There is therefore a logical interest in the development of antibacterial surfaces, serving to reduce microbial bioburden on these materials. By preventing the interaction with and adherence of bacterial cells to a surface, the initial stages of biofilm formation are disrupted, effectively removing the foundation which the bacterial biofilm requires. Antimicrobial polymeric coatings, fabrics, and paints are examples of approaches that have attracted interest to date [22]–[24].

This current study investigates the use of photosensitizer incorporation into polymers, with this approach intended to impart an antimicrobial and/or anti-adherent property to the material surface. Photosensitizers such as porphyrins and phenothiazines have been used clinically in photodynamic therapy (PDT) of malignancies [25]–[27], and have potential application in photodynamic antimicrobial chemotherapy (PACT), with photodynamic inactivation of MDR bacteria proving equally as effective as antibiotic-susceptible strains [28]–[31]. The mechanism of action of photodynamic therapy relies on the fact that photosensitizers are capable of reacting in the presence of visible light to produce cytotoxic effects. Phototoxic effects are initiated when, on absorption of an appropriate wavelength of light, the photosensitizer molecule is excited to the higher energy triplet state. This energy can be dissipated in one of two ways, *via* electron-transfer from the photosensitizer to a substrate, producing radical ions, which can react with oxygen, forming cytotoxic molecules such as superoxide, hydroxyl and lipid-derived radicals, or *via* direct energy transfer to oxygen, to produce the higher energy state singlet oxygen, which is highly reactive and can oxidize biological molecules such as proteins, nucleic acids and lipids, resulting in cytotoxicity [29], [31], [33].

Previously, we demonstrated how incorporation of photosensitizers into hydrogels can generate singlet oxygen on a biomaterial surface, with intended application in the design of infection-resistant medical devices [34], [35], [37]. Here, we generalise this concept, and demonstrate the facile production of a model two-layer poly(ethylene) (PE) film system, with one layer comprising sensitizer-incorporated PE, and the other a backing layer with the mechanical properties desired for the end application. Such a photodynamic, infection-resistant material may find broad application as coatings or covers for various inanimate objects commonly found in a hospital environment such as handrails, high-tech medical equipment (in particular touch-screens of IT devices), or materials for the manufacture of difficult-to-clean polymer surfaces such as coiled telephone cables or keypads. Incorporation of photosensitizer into PE by a hot-melt extrusion process, mechanical performance of sensitizer-incorporated polymer, leaching behaviour of sensitizer from the material, and antimicrobial properties of the material against a methicillin resistant strain of *S. aureus* and the Gram negative pathogen, *Escherichia coli*, upon light illumination are detailed. The results illustrate the viability of such materials as an effective general means for creating antimicrobial surfaces with the potential to control the spread of bacterial pathogens.

## Materials and Methods

### Materials

High-density poly(ethylene) (HDPE) was obtained from Q-Chem Ltd, Doha, Qatar (Marlex HHM TR-144, BN. 11100464). Low-denisty poly(ethylene) (LDPE) was obtained from LydonellBAsel Industries, Rotterdam, The Netherlands. Photosensitizers, cationic 5,10,15,20-Tetrakis(1-methyl-4-pyridinio)porphyrin tetra(p-toluenesulfonate) >97% (TMPyP) was obtained from TriPorTech GmbH, Lübeck, Germany. Neutral 5,10,15,20-Tetraphenyl-21H,23H-porphine 97% (TPP), Toluidine blue O (Tolonium chloride) 97% (TBO), and Methylene blue ≥82% (MB) were obtained from Sigma-Aldrich, Gillingham, UK, and were used without further purification.

### Extrusion of poly(ethylene) and sensitizer-incorporated poly(ethylene) materials

Extrusion was performed using a Dr Collin ZK 25 co-rotating twin screw extruder, with paired general purpose screws containing mixing section (25 mm dia; L/D ratio 36), and equipped with Dr Collin 250 mm slot die (coat hanger formation). Extrusion was controlled via a Dr Collin ECS-30 system and sheets were collected using a Dr Collin CR 136–350 chill-roll unit with three roll stack. Extruded materials were prepared in 1 kg batches containing pure PE or mixtures of either TMPyP, TPP, TBO, or MB and HDPE at sensitizer concentrations of 0.05% (0.50 g) and 0.40% (4.00 g) (w/w). The required weights of HDPE and sensitizer were mixed together until a consistent and even coating of HDPE with sensitizer was achieved. The extruder was pre-heated to 230°C along the screw and at the die. Pure HDPE was extruded initially in order to ensure the correct conditions had been obtained. Melt temperature was set at 222°C, screw speed at 60 rpm and pressure at the die was measured to be 114 bar. The chill roll was maintained at 110°C and roller speed was 1.2 metres per minute. Once a film of consistent quality and thickness was obtained, the hopper was emptied of remaining PE and was filled with the required sensitizer-containing PE mixture. Initial extrudate was discarded due to uneven mixing, resulting from the extrusion of the remainder of the pure PE resident in the screw, with collection beginning once pigmentation was uniform. Similarly, extrudate at the end of the batch was discarded as reduction in the available mass of the mixture resulted in reduced pressure, affecting the homogeneity of the film. The residence time of the sensitizer-PE mixture within the extruder was approximately 5 minutes.

### Production of twin layer sheets by platen press

Platen pressing was achieved using a Dr Collin P 200 P platen press, capable of maintaining a maximum temperature of 300°C and maximum pressure of 250 bar, with an effective operating area of 196×196 mm^2^. Sections of extruded sheets, one pure HDPE and a second sensitizer-incorporated HDPE, were cut to approximately 190×190 mm^2^ and placed one on top of the other inside a PTFE envelope. The envelope was placed on a tray and set in the platen press, pre-heated to 150°C, after which a five stage automated program was initiated, with temperature and pressure not exceeding 150°C and 70 bar respectively.

### Characterization of sensitizer-incorporated PE by UV-visible spectroscopy and confocal laser scanning microscopy

Confocal laser scanning microscopy was performed using a Leica DM RE upright microscope in conjunction with a Leica TCS SP2 system, and images were analyzed using Leica LAS AF imaging software. All images captured were 2048×2048 pixels, with a line average of 16 and were the average of 16 individual scans. The microscope pinhole was set at a 5.89 airy (600 µm) diameter. Reflectance images were recorded by setting the excitation beam wavelength to 488 nm and detecting emission in the range 480–500 nm. Fluorescence images required setting a suitable excitation wavelength and emission detection dependent on the photosensitizer under examination. For TPP, wavelengths used were 514 nm (excitation) and 600 nm –800 nm (emission), and for TMPyP wavelengths used were 514 nm (excitation) and 600 nm –720 nm (emission), while the emission wavelengths of MB and TBO lay outside the detection range of our instrumentation. Optical microscopy was therefore used to examine the distribution and homogeneity of MB and TBO within the polymer.

As a possible end application of these materials is antimicrobial covers for touch-screen devices, it is important to determine the transparency and optical clarity of the photosensitizer-incorporated films. UV-visible spectroscopy was performed using a Perkin Elmer Lambda 650 UV-visible spectrophotometer, to determine the optical transmittance of the materials in the visible region between 390–750 nm. This was achieved by attaching samples to the wall of a quartz cuvette, scanning this region of the electromagnetic spectrum, and determining mean transmission across this range of wavelengths. This analysis is used to determine the percentage of visible light that is transmitted through the photosensitizer-incorporated materials.

### Characterization of mechanical performance of sensitizer-incorporated materials

Mechanical analysis of films was performed on dumb-bell shaped samples (length 30 mm, thickness of narrow portion 2 mm) cut using a Ray-Ran hand-operated cutting press, and was tested using a Stable Micro Systems TA.XT plus texture analyzer, fitted with a 40 kg load cell. The dimensions of the narrow portion of the dumb-bell (width and thickness) were measured using a digital micrometer, and the samples were secured between the mobile upper and static lower clamps of the texture analyzer. The distance separating the upper and lower clamps was used to measure the gauge length of the sample. In order to test the sample, the upper clamp was raised at a speed of 50 mm.min^−1^, until sample fracture occurred. From the resultant stress–strain relationship, the mechanical properties of the samples (yield point, ultimate tensile strength (UTS), Young’s modulus and percentage elongation) were calculated. A minimum of five replicates of each sample were performed and the effect of incorporation of varying concentrations of both photosensitizers on the mechanical properties of PE was assessed for statistical significance using a one-way ANOVA, with post-hoc comparisons made using Tukey’s HSD test. Significance was denoted by a value of p<0.05.

### Characterization of leaching behaviour

Photosensitizer-incorporated samples (30×20 mm) were cut and pressed firmly 1000 times with one clean, washed finger. Adherent photosensitizer was washed from the finger by immersing in 10 mL deionised water for 30 seconds. Solutions were analyzed using a Perkin Elmer Lambda 650 UV-visible spectrophotometer; the concentration of photosensitizer in solution was determined via the calculated molar extinction coefficient, (ε) at λ_max_, of solutions of known concentrations. Calculated ε values are as follows: TBO 39,750 mol^−1^ dm^−3^ cm^−1^ (λ_max_ 610 nm); TPP 406,750 mol^−1^ dm^−3^ cm^−1^ (λ_max_ 420 nm); MB 74,028 mol^−1^ dm^−3^ cm^−1^ (λ_max_ 664 nm); TMPyP 193,000 mol^−1^ dm^−3^ cm^−1^ (λ_max_ 446 nm). Using the same sample, the materials were pressed a further 1000 times, repeating the procedure as before, and continuing to touch samples in increments of 1000, up to 10,000 times. Analyses were carried out in triplicate.

Photosensitizer-incorporated samples (75×50 mm) were cut and wiped 50 times with a medical wipe moistened in a 1% (w/v) solution of non-ionic surfactant, Tween 20, as a model for typical hospital surface cleaning products. Leaching of the highly-colored photosensitizer by this surface cleansing procedure was assessed by examination of the degree of staining of the medical wipe. Using the same sample, the materials were wiped a further 50 times, repeating the procedure as before, and continuing to clean samples in increments of 50, up to 500 times. Analyses were repeated in triplicate.

### Characterization of antimicrobial behaviour

Bacterial adherence to materials was tested using methicillin-resistant *Staphylococcus aureus* (MRSA) ATCC 33591; bacteria were grown aerobically at 37°C in Müller–Hinton Broth (MHB) for 18 hours. During log phase of growth, the broth culture was centrifuged at 3000 rpm for 12 minutes, the cell pellet re-suspended in phosphate buffered saline (PBS). The suspension was diluted, such that the optical density was 0.3 at 540 nm (approx. 2.0×10^8^ cfu/mL). The suspension was serially diluted in PBS, to form the final inoculum (approx. 4.0×10^5^ cfu/mL). Sensitizer-loaded samples and controls (blank HDPE without sensitizer) were cut to 15×10 mm. Drops of bacterial inoculum (60 µL – approx. 1.6×10^4^ cfu/cm^2^) were placed into a sterile petri dish, after which each drop was covered with a single sensitizer-loaded or control sample. Petri dishes containing inoculated samples were inverted, in order to facilitate light irradiation of the polymer surface in contact with the bacteria, and placed under two 230W halogen bulbs situated 24 cm from dishes, providing 1340 µW light across the sample surface, for 2 hours. Samples are held in place against the base of the inverted petri dish by adhesive capillary force.

Post-illumination, samples were removed from petri dishes and placed in 10 mL PBS, inverting constantly for 30 seconds, to remove non-adherent bacteria; samples were then transferred to individual bijoux bottles containing 2 mL Quarter Strength Ringers Solution (QSRS). Samples were sonicated for 10 minutes in order to remove and suspend adherent bacteria, using a Branson 3510 ultrasonic cleaner providing a fixed puissance of 42 KHz. The number of surviving microorganisms were determined by spread plating on Müller–Hinton Agar (MHA) plates. Plates were allowed to dry, inverted and incubated at 37°C, 50% RH for 18–24 hours. Testing was performed using five replicates of each sensitizer-loaded material (at both high and low concentrations), along with corresponding polyethylene control. Lead materials identified as effective against MRSA were then carried forward for further testing with Gram-negative *Escherichia coli* NCTC 8196. These lead materials (MB 0.4%, TBO 0.4%, and TMPyP 0.4%) were tested against *E. coli* as per methodology described above. Statistical significance was determined against dark HDPE control and was carried out using a two-tailed student’s t-test, p<0.05.

## Results

### Distribution of sensitizer in materials and optical transmittance of sensitizer incorporated films

**Figure 1:**
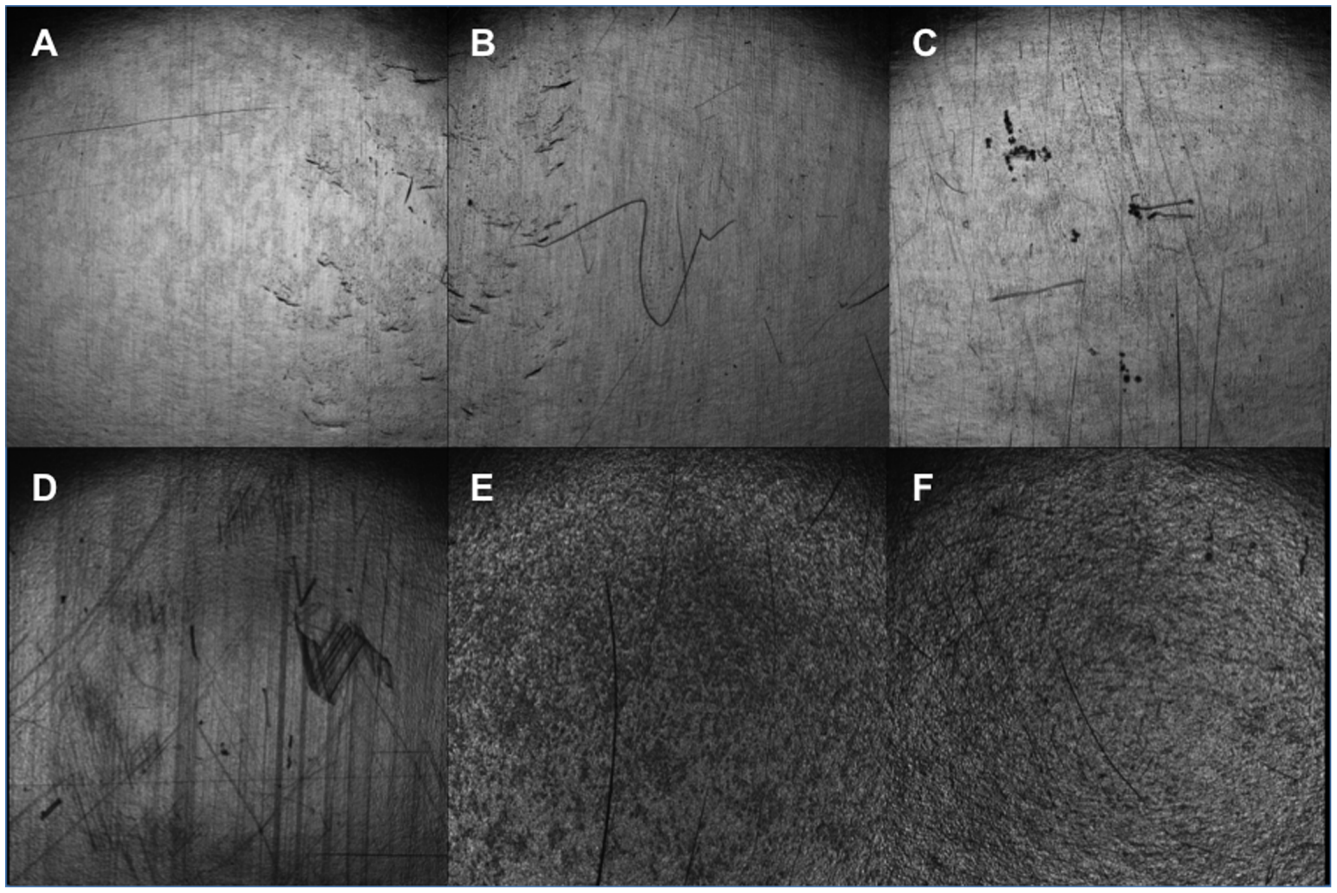
CLSM reflctance image of (A) HDPE control, (B) 0.40% TPP-HDPE, (C) 0.40% TMPyP-HDPE surfaces, (D) HDPE control, (E) 0.40% MB-HDPE, (F) 0.04% TBO-HDPE. All images represent an area measuring 3.75 mm x 3.75 mm.

HDPE sheets containing varying concentrations of sensitizers were subject to examination by confocal laser microscopy. Samples were first viewed in reflectance mode, in order to provide images of the material surfaces. No distinct visual alterations to the polymer surface were seen upon addition of TPP to the HDPE mixture, however, inspection of TMPyP incorporated HDPE appears to show some aggregates at the material surface; there also appears to be some modification to the polymer surface upon addition of the higher concentrations of MB and TBO, likely due to the presence of aggregates at the material surface, compared with the relatively smooth surface of the pure HDPE, as illustrated in Figure 1.

**Figure 2:**
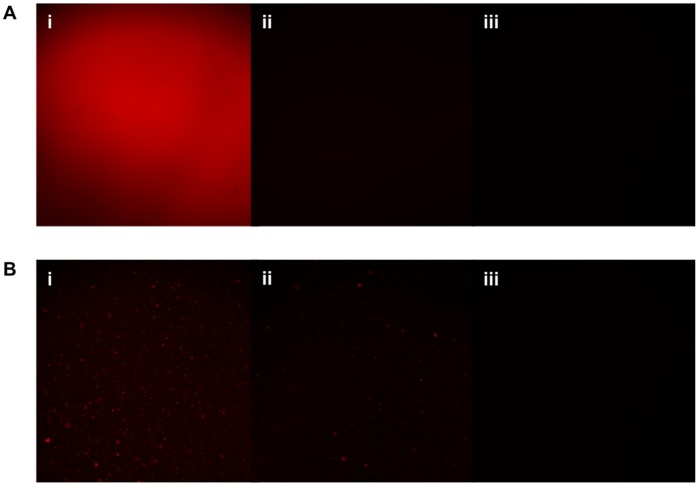
CLSM fluorescence micrographs of (A) TPP-HDPE at (i) 0.40%, (ii) 0.05%, (iii) 0% TPP (control) and of (B) TMPyP-HDPE at (i) 0.40%, (ii) 0.05%, (iii) 0% TMPyP (control). All images represent an area measuring 1.5 mm x 1.5 mm.

The use of fluorescence imaging allowed visualization of the sensitizer throughout the materials. At both 0.40% and 0.05% TPP loading, an even fluorescence signal was observed on the surfaces of the samples, indication that the photosensitizer has been well mixed with the polymer and has produced a largely homogenous surface, as displayed in Figure 2. However, fluorescence imaging of the surface of TMPyP incorporated HDPE revealed incomplete mixing of the photosensitizer with the material, at both the 0.05% and 0.40% concentrations (Figure 2).

**Figure 3:**
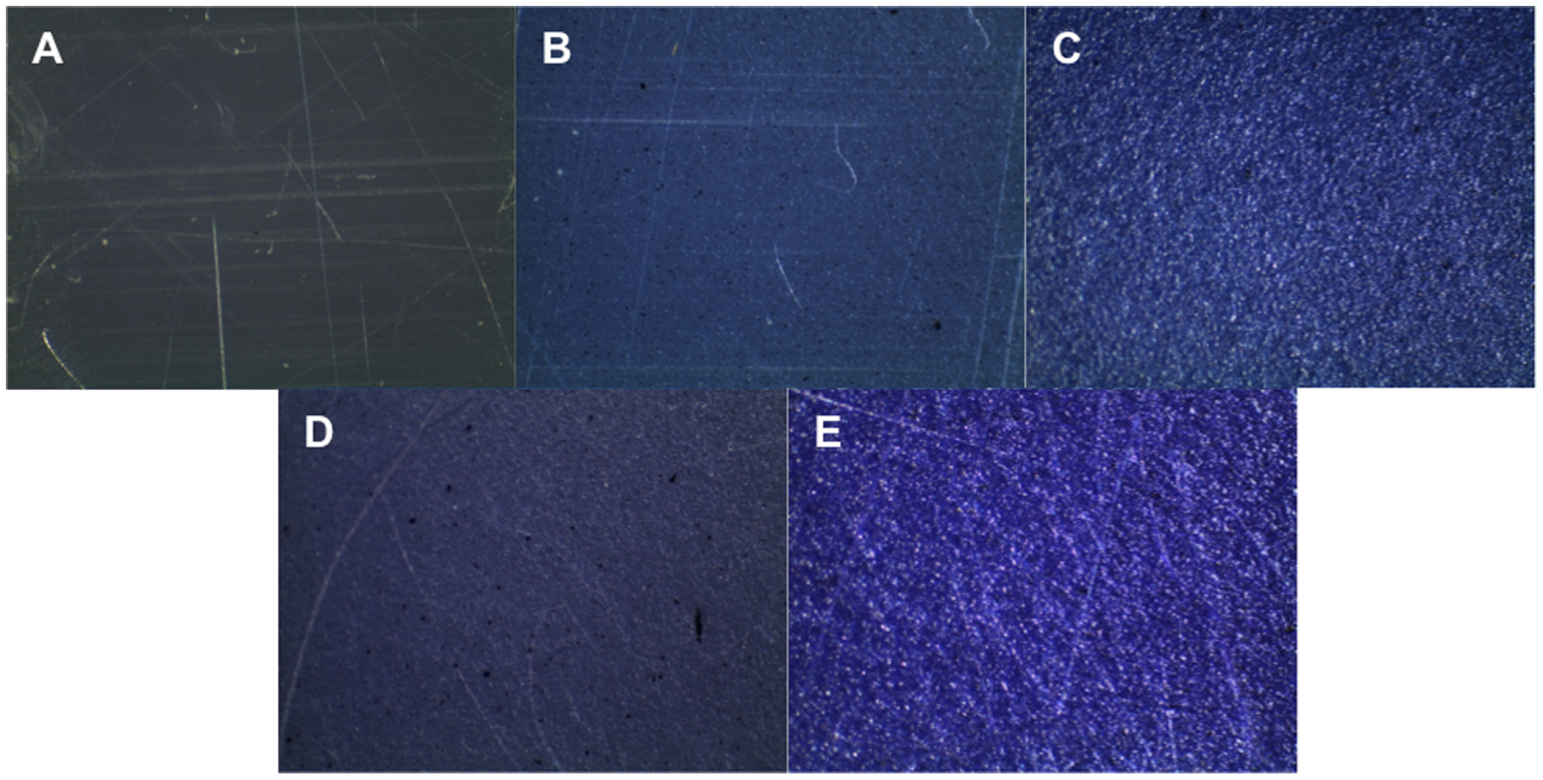
Optical microscope images of (A) PE, (B) 0.05% MB-PE, (C) 0.40% MB-PE, (D) 0.05% TBO-PE, and (E) 0.40% TBO-PE. All images represent an area measuring 1.0 mm x 1.0 mm.

As fluorescence imaging of materials containing TBO and MB was not possible, optical microscope images were collected and revealed a relatively even colouring with minimal alteration in the surface compared to HDPE; however, the images show small darkened spots indicating non-homogeneity and incomplete mixing of the sensitizers (Figure 3).

UV-visible spectroscopic analysis of photosensitizer-loaded samples was performed to determine the transmittance of light through the sample, in order to give a measure of optical clarity and transparency. The mean transmittance of pure polyethylene film, across the visible range (390 nm–750 nm), was 68%. Compared with this, the transmittance of low concentration porphyrin-incorporated materials (0.05% TPP and TMPyP) was approximately 49%, in both cases. Samples containing 0.4% TMPyP exhibited transmittance of 37%, while 0.4% TPP reduced transmittance to 33%. For phenothiazine incorporated films, the transmittance of low concentration materials (0.05% TBO and MB) was approximately 46% and 44%, respectively. Samples containing 0.40% MB exhibited transmittance of 28%, while 0.40% TBO reduced transmittance to 14%.

### Mechanical performance

**Figure 4:**
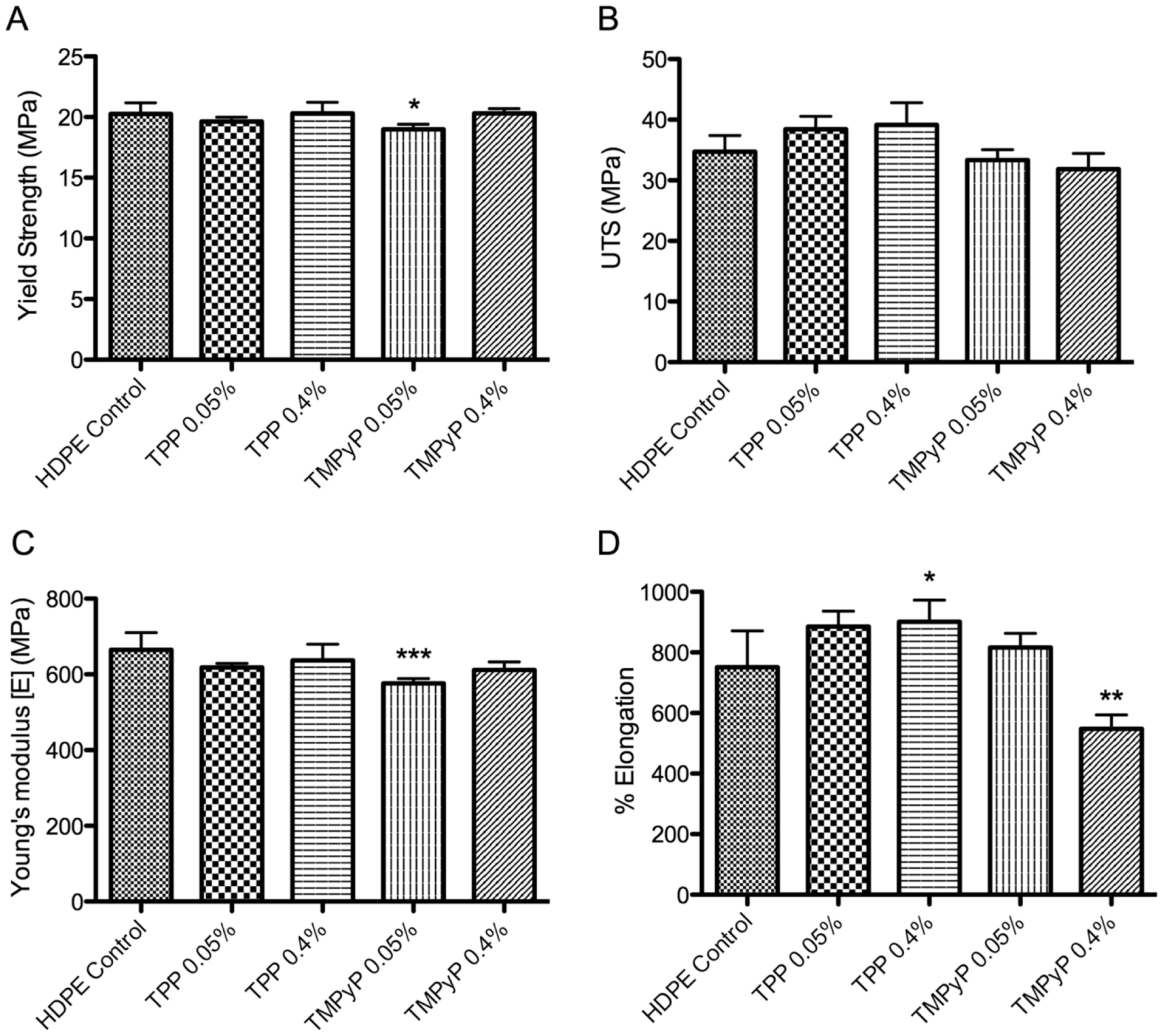
Effect of incorporation of TPP or TMPyP on extruded HDPE sheets (at 0.05 or 0.40% w/w) on: (A) yield strength, (B) ultimate tensile strength, (C) Young’s Modulus and (D) percentage elongation at break. * indicates level of significance from HDPE control, graph displays means+standard deviation.

**Figure 5:**
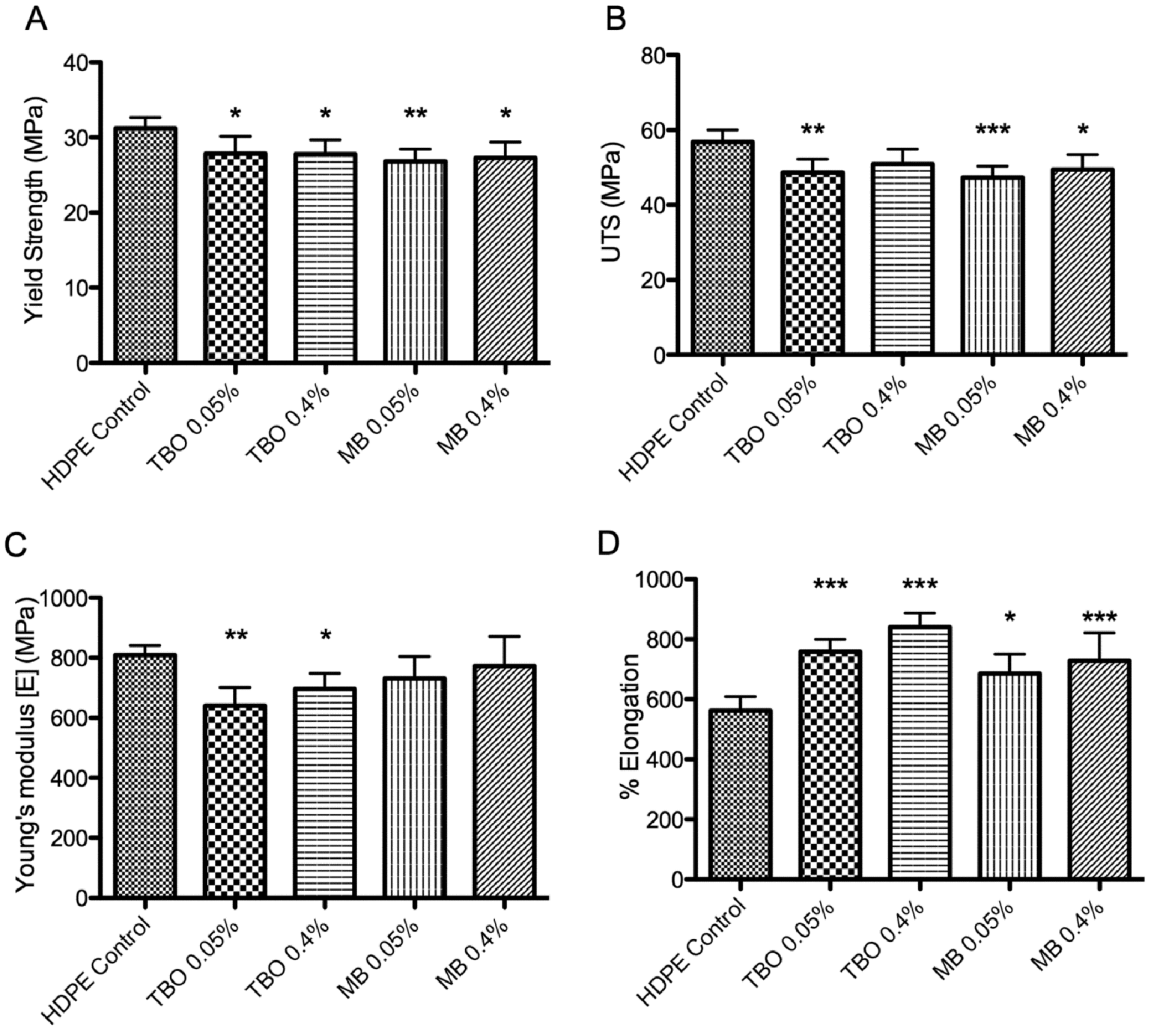
Effect of incorporation of TBO or MB on extruded HDPE sheets (at 0.05 or 0.40% w/w) on (A) yield strength, (B) ultimate tensile strength, (C) Young’s Modulus and (D) percentage elongation at break. * indicates level of significance from HDPE control, graph displays means+standard deviation.

Mechanical testing of prepared HDPE samples, containing either TPP, TMPyP, MB or TBO at concentrations of 0.05 or 0.40% (w/w), was performed and the results compared to the mechanical properties of pure HDPE. Using the data collected, four measures of the mechanical performance of the materials were calculated – yield strength, ultimate tensile strength (UTS), Young’s Modulus, and percentage elongation, which are shown in Figure 4 (porphyrin-based sensitizers, TPP and TMPyP) and Figure 5 (phenothiazine-based sensitizers, TBO and MB).

These results indicate that incorporation of TPP at either concentration causes no significant effect on the yield strength of PE (20.3±0.90 MPa vs. 19.6±0.36 (0.05%) and 20.3±0.92 (0.40%)). Incorporation of TMPyP at concentrations of 0.05% causes a statistically significant reduction in the yield strength of the material (19.0±0.41 MPa); while HDPE with a TMPyP-loading of 0.40% produces no significant difference in yield strength from that of PE (20.3±0.35 MPa). TBO at either concentration causes a significant reduction on the yield strength of PE (31.1±1.47 MPa vs. 27.9±2.27 (0.05%) and 27.8±1.94 (0.40%)). Similarly, incorporation of MB at concentrations of 0.05% and 0.40% also causes a significant reduction in the yield strength of the material (26.8±1.5 and 27.3±2.18, respectively).

Evidence suggests that TPP may have a strengthening effect on HDPE, successively increasing UTS as concentration is increased from 0.05% to 0.40% (34.8±2.7 MPa vs. 38.5±2.1 (0.05%) and 39.2±3.7 (0.40%)). Conversely, results suggest the TMPyP reduces UTS of HDPE, with increasing concentration (33.4±1.7 (0.05%) and 31.9±2.6 (0.40%)). However, statistical analysis finds no significant difference between photosensitizer-incorporated HDPE and standard HDPE sheets; similar analysis indicates TPP-loaded PE sheets (both concentrations) exhibit significantly greater UTS than the TMPyP counterparts. TBO and MB both affect ultimate tensile strength. Incorporation of MB at either concentration resulted in significant reduction in ultimate tensile strength (56.9±3.22 MPa vs. 47.2±3.21 (0.05%) and 49.5±4.00 (0.40%)). In a similar manner, the presence of a 0.05% loading of TBO results in a significant reduction in ultimate tensile strength; while, although not significant, 0.40% TBO also reduced tensile strength (48.6±3.73 (0.05%) and 51.0±3.9 (0.40%)).

Addition of TPP produces no significant change in the Young’s modulus from that of PE (665±45 MPa (PE) vs. 619±10 (0.05%) and 637±42 (0.40%)); however inclusion of 0.05% TMPyP in PE sheets results in significant reduction in Young’s modulus (576±13 MPa), indicating increased elasticity and reduced stiffness of these samples. Higher concentrations of TMPyP (0.40%) produce no significant change compared with PE (611±21 MPa). Addition of 0.05% TBO produces significant alteration of the Young’s modulus of PE (808±34 MPa vs. 641±60); addition of 0.40% TBO also results in significant reduction (695±53 MPa), however the reduction observed is not as great and suggests that on increasing TBO loading further, materials may return to similar levels to that of PE. Inclusion of MB in PE sheets results in a similar pattern to that of TBO, however reductions in Young’s moduli (731±75 MPa (0.05%) and 772±95 (0.40%)) are not considered statistically significant. It should be noted that standard deviation of Young’s moduli of photosensitizer-incorporated PE samples are greater than that observed for PE suggesting that inclusion of photosensitizers has resulted in greater variability with regard to this mechanical property.

Use of TPP, initially, produces moderate, but statistically insignificant, increase in elongation from that of PE (751±120% vs. 885±51 (0.05%)); however increasing concentration to 0.40% significantly increases elongation (901±72%). Similarly, 0.05% TMPyP results in no significant changes in elongation (816±46%); however at concentrations of 0.40%, TMPyP significantly reduces the percentage elongation of the sample (548±46%). Use of TBO produces significant increases in elongation, with increasing concentration, from that of PE (563±48% vs. 756±42 (0.05%) and 840±47% (0.40%)). Similarly, MB inclusion also results in significant increases in elongation (684±68% (0.05%) and 728±94% (0.40%)).

### Leaching behaviour

Touch testing was performed on samples containing TPP, TMPyP, TBO, and MB, all at a concentration of 0.4%. It was observed that after 10,000 touches of the TMPyP, MB and TBO samples, no photosensitizer had leached from the material. A small amount of leaching was observed from TPP-loaded HDPE, equating to 0.0045% of the total TPP in the sample tested.

Leaching of photosensitizer from the material during surface cleansing was assessed by wiping the material surface with a medical wipe moistened in 1% (w/v) Tween 20. Slight staining of the medical wipe was evident in the case of TMPyP, TPP and MB, although the amount could not be quantified due to the difficulty in extracting the photosensitizer from the wipe into solution for UV-visible spectroscopic analysis. For TBO surface cleansing did not result in the staining of the medical wipe, indicating that for this photosensitizer, leaching was negligible or failed to occur.

The experimental design used in these leaching tests reflects the environment the materials would experience in their intended end application. These results indicate that negligible leaching would occur during normal skin contact or wipe cleaning with detergent.

### Characterization of antimicrobial behaviour

**Figure 6:**
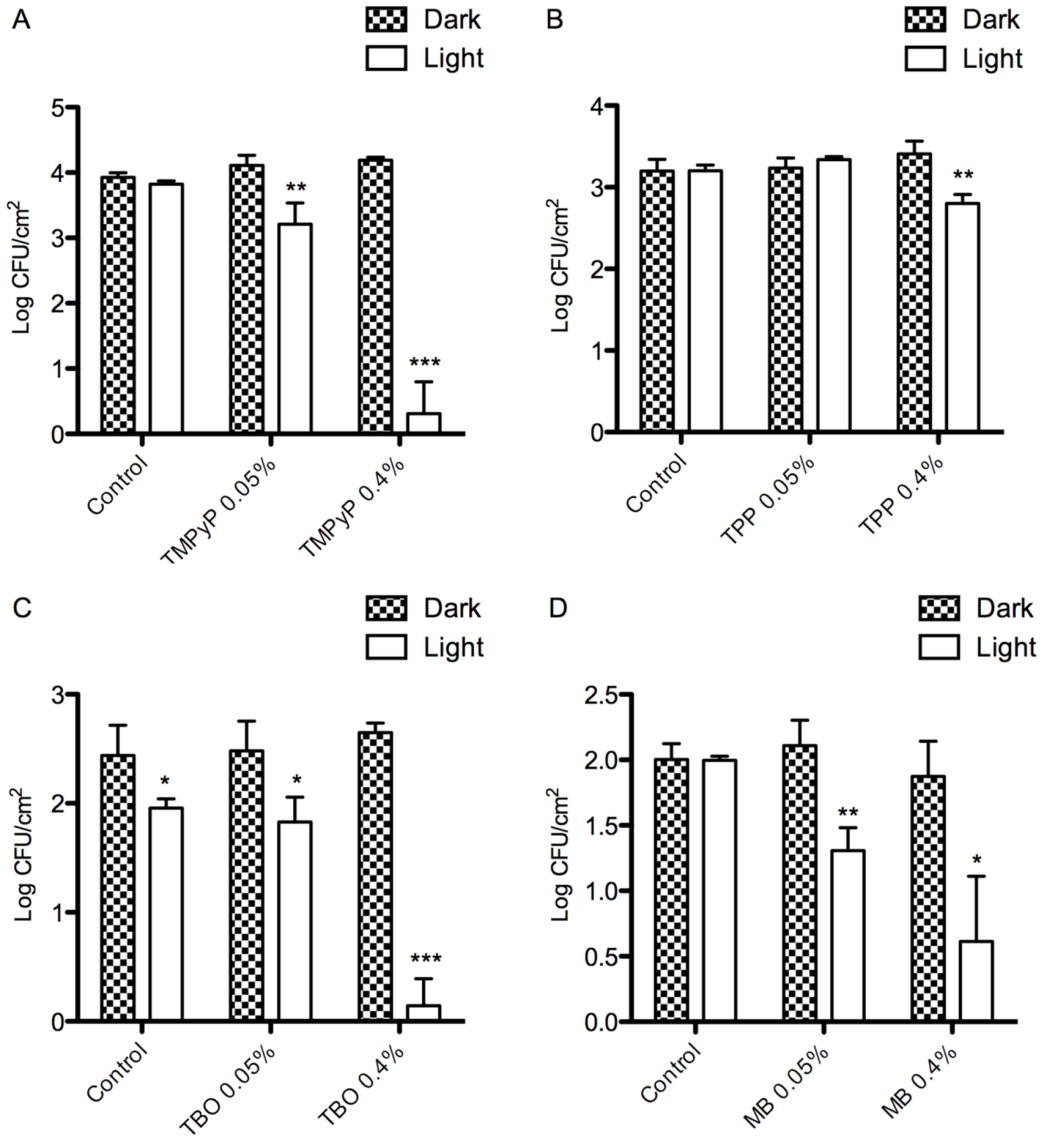
Reduction in adherence of viable MRSA on the surfaces of materials loaded with (A) TMPyP, (B) TPP, (C) TBO and (D) MB. Log CFU/cm^2^ adhered bacteria were enumerated on control materials (HDPE without photosensitizer), and test samples in both dark and light-irradiated conditions. * indicates level of significance from HDPE dark control, graph displays means+standard deviation.

**Figure 7:**
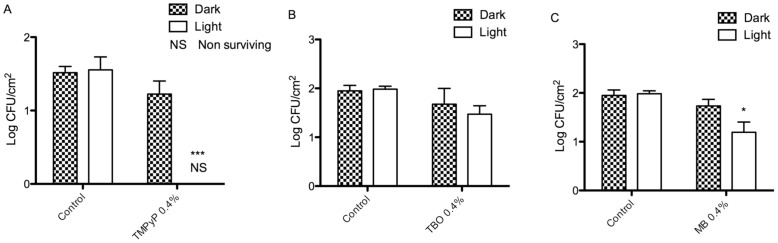
Reduction in adherence of viable *E. coli* on the surfaces of materials loaded with (A) TMPyP, (B) TPP, (C) TBO and (D) MB. Log CFU/cm^2^ adhered bacteria were enumerated on control materials (HDPE without photosensitizer), and test samples in both dark and light-irradiated conditions. * indicates level of significance from HDPE dark control, graph displays means+standard deviation.

Antimicrobial adherence was performed by inoculating samples with MRSA and *E.coli* and determining the number of viable bacteria after two hours, in either light or dark conditions. Only lead materials that demonstrated good antibacterial activity against Gram-positive MRSA were taken forward to testing against Gram-negative *E.coli*. Gram-negative bacteria are widely considered to be more tolerant to photoinactivation than their Gram-positive counterparts [29], [36]. Reduction in bacterial adherence on light irradiation, when compared to identical material samples inoculated in the dark, and additional controls of non-photosensitizer incorporated materials in both light and dark conditions, are shown in Figures 6 and 7.

Figure 6 illustrates the results obtained against MRSA. It is clear that the greatest log-cycle reduction in colony-forming units (CFU) per square centimetre material are observed for those samples containing the higher (0.4%) concentration of photosensitizer. HDPE containing TPP 0.4% displayed the least anti-microbial activity, with a 0.40 log-cycle reduction in MRSA adherence when compared to dark HDPE control. MB 0.4%, TBO 0.4% and TMPyP 0.4% showed a 1.39, 2.30, and 3.62 log-cycle reduction against MRSA adherence respectively.

As shown in Figure 7, *E.coli* were inherently less adherent than MRSA on all materials, including the HDPE controls without photosensitizer. HDPE containing 0.4% TMPyP displayed excellent anti-bacterial and anti-adherent characteristics, with no viable organisms detected after light irradiation, equating to a 1.51 log-cycle reduction in adhered *E.coli,* when compared to HDPE dark control. MB 0.4% and TBO 0.4% did not perform as favourably, resulting in a 0.75, and a 0.47 log-cycle reduction in adherence, respectively.

## Discussion

Several photosensitizing compounds, namely TMPyP, TPP (phorphyrin-based), TBO and MB (phenothiazine dyes), were successfully incorporated into high density poly(ethylene) (HDPE) films by a hot-melt extrusion process, and incorporated into a twin-layer polymer sheet by platen press. Our aim is to develop photodynamic antimicrobial surfaces that may be used in a healthcare environment to prevent the spread of nosocomial infection. The antimicrobial surface is the result of the generation of reactive oxygen species (ROS) due to illumination of photosensitizer molecules present in the polymer film, as demonstrated by our earlier work on related materials [28]. These ROS react indiscriminately with components of the bacterial cell, such as nucleic acids, proteins and lipids, eventually causing cell death [29], [31]–[33].

The hot-melt extrusion process was successful in incorporating photosensitizer into HDPE films, although the homogeneity of photosensitizer distribution within the film appears to vary depending on the photosensitizer used. CLSM fluorescence microscopy revealed favourable homogenous distribution of TPP within the extruded polymer, and this is likely due to the neutral hydrophobic properties of TPP allowing for complete miscibility with the poly(ethylene). The other sensitizers used, TMPyP, TBO and MB, all show some evidence of photosensitizer aggregation and incomplete mixing, with the phenothiazines TBO and MB to a lesser extent than TMPyP. It is desirable to maintain a smooth material surface, as an increase is surface roughness or irregularity may promote adhesion of bacteria. Figure 1 shows that at the higher photosensitizer concentrations employed (0.40%), a smooth, regular surface was maintained in films incorporating the porphyrin-based TPP and TMPyP while TBO and MB did display evidence of surface modification, however the effects of this on the ability of bacteria to adhere to the material surface will only be evident through microbiological testing.

Characterization of mechanical properties of the prepared films by determination of yield point, ultimate tensile strength, Young’s modulus and percentage elongation measurements reveal that incorporation of TPP and TMPyP have a minimal effect on the mechanical characteristics of the HDPE films, while incorporation of TBO or MB have a slight negative effect, suggesting that these sensitizers may adversely effect the quality of the material. However, if TBO or MB were incorporated into the twin layer system as the minor layer, the overall effect is likely negligible.

UV-visible spectroscopy demonstrated that incorporation of photosensitizer into the HDPE films has a negative effect on the optical clarity of the materials given that mean transmission over the visible range (390 nm –750 nm) is reduced in comparison to control HDPE, especially at the higher concentration of photosensitizer used. The implications of this observation depend on the intended purpose of the material. For example, a cover for a touch-screen computer would require the material to have good optical transmittance. It should be noted, however, that in this study the thickness of the photosensitizer-incorporated material is approximately 200 µm and with a different manufacturing process, such as blown-film extrusion, this may be reduced to 25 µm, and hence it is expected that optical transmittance of the photosensitizer incorporated films would improve dramatically.

The study of leaching behaviour from materials *via* touch-testing shows that TMPyP, TBO and MB are not removed from the material surface by this mechanism, while minimal amounts of TPP are lost after 10,000 touches. This favourable leaching behaviour is important should these materials be used as covers for equipment such as touch-screen covers, keypads or handrails.

Antimicrobial testing showed that the most effective photosensitizer for incorporating into antimicrobial HDPE films was TMPyP at the high concentration of 0.40%. This material displayed a 3.61 log-cycle reduction in viable MRSA, and 1.51 log-cycle reduction in *E. coli* after two hours light irradiation at 1340 µW/cm^2^ per sample, using halogen bulbs as a light source. The ability of the TMPyP-incorporated HDPE in achieving a greater than one log-cycle reduction of a Gram-negative bacterium is encouraging, since the Gram-negative outer membrane functions as a barrier, protecting the cell and rendering it more resilient to photodynamic inactivation. It is the polycationic nature of the TMPyP molecule that imparts an advantage over the neutral TPP photosensitizer, as the cation is necessary for interaction and disruption of the outer membrane, so making inactivation with ROS possible [36].

The white light generated by the halogen bulb light source is inexpensive and representative of lighting conditions found in typical artificially-illuminated indoor environments, meaning that photosensitizer incorporated materials would not require any special conditions under which to produce their antimicrobial effect, as long as the environment in which they are used is adequately illuminated. Alternatively, for specific decontamination purposes, such as during deep-cleaning of hospital operating theatres, specialized lighting could be installed to emit at higher intensity, and at a wavelength specific for the excitation of the photosensitizer employed, for efficient generation of ROS and the resulting bactericidal effects.

This study, aimed at controlling the microbial bioburden and infectious reservoir on inanimate, everyday objects, builds upon our previous work in the development of photo-activated, antimicrobial polymers for the prevention of medical device associated infection [34], [35], [37], thereby expanding scope of photo-active materials for potential infection control applications in a healthcare setting. Other published studies have documented the incorporation of photosensitizing compounds into various materials with the aim of generating anti-microbial or anti-infective surfaces. For example, Krouit *et. al.* (2008) incorporated a cationic porphyrin into a cellulose-based material [38]. However, the microbiological assays used in their work make it difficult to conclude if the reduction in viable bacteria was due to contact of sessile bacteria with the material surface, or release and diffusion of porphyrin into the nutrient agar used, in a similar fashion to antibiotic testing ‘zone of inhibition’ assays. Conversely the methodology used in our present study is specific against assessing the photo-inactivation of sessile, surface immobilised, adherent bacteria.

Our photosensitizer incorporated HDPE films display similar antibacterial efficacy as a photoactive cotton fabric, developed by Ringot *et. al.* (2011). This group also demonstrated the inherent difficulty of photo-inactivation of Gram-negative species in comparison to Gram-positive. The cotton-based fabrics documented in their study showed up to 5 log-cycle reductions in viable *S. aureus*, but only an approximate 1 log-cycle reduction in *E.*coli [39]. These figures are comparable to those we have obtained in our present work.

While we have focused on the antibacterial activity of our developed materials, future work shall investigate their ability to resist colonization with other pathogens, such as yeasts. Alvarez *et. al.* (2012) have reported successful inactivation of *Candida albicans* using polysilsesquioxane films doped with porphyrin [40] ^.^

## Conclusions

This study demonstrates a general method to manufacture light-activated antimicrobial surfaces through the incorporation of photosensitizers into polymer films, specifically exemplified using high density poly(ethylene) showing high levels of antimicrobial behaviour, even to resistant strains such as MRSA and Gram-negative organisms in the presence of visible light. The use of such materials in the healthcare setting as coatings or coverings of inanimate objects such as keypads, touchscreens or handrails, may remove these surfaces as reservoirs of nosocomial pathogens. This may assist in the prevention of hospital-acquired infections, which are currently detrimental to the morbidity and mortality of vulnerable patients, and also have a significant financial impact on the resources of modern healthcare systems and institutions.
